# Sundowning in Dementia: Clinical Relevance, Pathophysiological Determinants, and Therapeutic Approaches

**DOI:** 10.3389/fmed.2016.00073

**Published:** 2016-12-27

**Authors:** Marco Canevelli, Martina Valletta, Alessandro Trebbastoni, Giuseppe Sarli, Fabrizia D’Antonio, Leonardo Tariciotti, Carlo de Lena, Giuseppe Bruno

**Affiliations:** ^1^Department of Neurology and Psychiatry, “Sapienza” University of Rome, Rome, Italy

**Keywords:** sundowning, sundown syndrome, behavioral disruptions, neuropsychiatric symptoms, dementia, behavioral and psychological symptoms of dementia, circadian rhythm

## Abstract

Sundowning means the emergence or worsening of neuropsychiatric symptoms (NPS) in the late afternoon or early evening. This syndrome has been recognized since a long time in the field of dementing illnesses and is well known among most of health-care providers involved in the assistance of people with dementia. Indeed, it represents a common manifestation among persons with dementia and is associated with several adverse outcomes (such as institutionalization, faster cognitive worsening, and greater caregiver burden). Its occurrence and phenotypic characteristics may be influenced by diverse neurobiological, psychosocial, and environmental determinants. Moreover, it may pose diagnostic challenges in relation to other common causes of behavioral disruptions. Beside these considerations, this phenomenon has so far drawn limited clinical and scientific interest compared to other specific NPS occurring in dementias, as indicated by the lack of commonly agreed definitions, specific screening/assessment tools, and robust estimates on its prevalence. Accordingly, no randomized controlled trial specifically investigating the effectiveness of pharmacological and non-pharmacological strategies in managing this condition among demented patients has been yet conducted. In the present narrative review, we present and discuss available evidence concerning sundowning occurring in people with dementia. A special focus is given to its definitions, pathophysiological determinants, and clinical relevance, as well as to the clinical and therapeutic approaches required for its management in the daily practice.

## Introduction

Neuropsychiatric symptoms (NPS) represent core features of dementias, occurring in the overwhelming majority of cases and being highly burdening for patients and families. Moreover, they constitute a major determinant for health-care expenditures (1, 2). The clinical and research approach to these manifestations is extremely challenging. Indeed, NPS are characterized by a marked interindividual variability. Their prevalence and severity change over the course of the disease. Moreover, multiple interacting variables and pathophysiological mechanisms may influence their occurrence and phenotypic expression (3). These aspects have been frequently hindering the application of standardized clinical and analytic approaches to NPS, as well as the identification of targeted pharmacological interventions (4).

A condition that properly mirrors the relevant complexity of the neuropsychiatric manifestations of dementia is represented by the “sundown syndrome,” that is, the emergence or worsening of NPS in the late afternoon or early evening. This entity has been recognized since a long time in the field of dementing illnesses (5) and is well known among most of health-care providers involved in the assistance of persons with dementia. Curiously, even many ordinary people who have personal experiences or acquired knowledge about neurodegenerative diseases are aware of this “bizarre” phenomenon. Nevertheless, the sundown syndrome has so far drawn limited clinical and scientific interest compared to other specific NPS and behavioral disturbances occurring in dementias (e.g., apathy, depression, psychotic symptoms). As a proof, the available data concerning its prevalence (mostly obtained in institutional/residential settings) are scarce and markedly discordant in the literature. Moreover, no randomized controlled trial (RCT) specifically investigating the effectiveness of pharmacological and non-pharmacological strategies in managing this condition among demented patients has been yet conducted. Several aspects may account for this low attention, such as the lack of commonly agreed definitions, the absence of specific screening and assessment tools and the multiplicity of factors that may trigger or affect its occurrence.

In the present narrative review, we present and discuss the evidence collected so far concerning the clinical characteristics and relevance of the sundown syndrome, its pathophysiological determinants and its pharmacological and non-pharmacological management. Special attention will be also dedicated to the methodological issues that are still limiting and hampering the clinical and research approach to this phenomenon.

## Definition of Sundowning

A clear and univocal definition of the sundown syndrome (often referred to as “sundowning”) has not been yet achieved. These terms are broadly used to describe a set of NPS occurring in elderly patients at and/or after the time of sunset. Diverse conceptual aspects have hampered the formulation of a unique definition. First of all, some authors have restricted the adoption of the sundowning construct to only people with dementia, whereas others have described this phenomenon also among cognitively intact elderly individuals (even if with lower frequency) (6). A second element of heterogeneity relies in the clinical manifestations included in the sundown syndrome. Indeed, some definitions refer to the onset or worsening of specific NPS [agitation in particular (7)], while others more widely include any behavioral and psychological disturbance. In this regard, these behaviors may consist of a wide variety of symptoms such as anxiety, agitation, aggression, pacing, wandering, resistance, screaming, yelling, visual and auditory hallucinations, and so forth (8). Several conceptualizations also incorporate the exacerbation of cognitive symptoms and confusion (7), thus rendering more subtle the distinction with delirium. Finally, a relevant disagreement concerns the timeframe when increased symptoms should occur to configure a sundowning. Most of available definitions include only the occurrence or worsening of behavioral disturbances during the late afternoon and early evening (9, 10), whereas other researchers also consider NPS occurring throughout the night (11) or more vaguely emerging with darkness (12). It is noteworthy that some authors even question the existence of the sundown syndrome, hypothesizing that symptoms occurring during the entire day may simply be more burdening for nursing staff and caregivers in late afternoon and evening (13).

As result of the lack of consensus around the definition of sundowning, differently from other neuropsychiatric syndromes occurring among elderly individuals (e.g., delirium), no standardized criteria have been formulated for its diagnosis [e.g., the sundown syndrome does not appear in the recent fifth revision of the Diagnostic and Statistical Manual of Mental Disorders (14)].

## Relevance of Sundowning

Beyond the above-mentioned need of agreed definitions, sundowning is a relevant clinical phenomenon. First of all, it represents a common manifestation among people with dementia. The available studies on the topic have reported prevalence rates widely ranging between 2.5% and 66% depending on the study setting, the adopted operationalizations, and the underlying clinical diseases (6, 15). Sundowning has been observed to represent the second most common type of disruptive behavior in institutionalized patients with dementia after wandering and has been frequently described as “endemic” in nursing homes hosting cognitively impaired older subjects (8, 16). At the same time, it has also been commonly described among community-dwelling individuals with dementing illnesses [e.g., in the 66% of patients with Alzheimer’s disease (AD) living at home (17)]. Based on the data from the Alzheimer’s Association, as many as 20% of patients diagnosed with AD may experience a sundown syndrome (18). This phenomenon has been also reported in the context of non-AD dementias (e.g., vascular dementias, frontotemporal dementias, Lewy body dementias). Nevertheless, the scarcity of epidemiological data does not allow to properly explore the different prevalence of sundowning in the specific dementia conditions. Along the same lines, there are no consistent data concerning its prevalence according to the age, sex, and race of patients, whereas the severity of cognitive impairment has been repeatedly recognized as important predisposing factor in its development (7, 8). Finally, some data suggest a different seasonal occurrence of sundowning, with an observed higher incidence in the fall or winter months (10).

The relevance of the sundown syndrome also relies on its association with several adverse outcomes among people with dementia and their families. Sundowning has been indicated as a common cause of institutionalization of older demented patients. It poses a significant social and economic burden in terms of recurrent hospitalizations, prolonged hospital stay, and functional decline (1, 15), and it has also been associated with a faster progression of cognitive worsening in AD (19). Moreover, a relationship between its occurrence and the perceived stress of caregivers assisting AD patients has been demonstrated (17). Behavioral disruptions in the late afternoon or in the evening may pose a further specific challenge to caregivers who have to handle such symptoms at the end of the day, when they may be particularly fatigued. Some sundown behaviors may prevent patients from sleeping well, making them more likely to wander, thus increasing the risk for caregiver sleepiness and burnout (20, 21). A “stressed caring” of a burdened and fatigued caregiver may, in turn, lead to act wrong management strategies and increase the likelihood of an exacerbation of NPS exhibited by the patient, thus triggering a potential dangerous loop (22).

Based on these considerations, there is a growing consensus around the need of achieving a better understanding of sundowning and developing effective strategies for its management.

## Pathophysiology of Sundowning

The pathophysiology of sundowning is, to date, poorly defined since no causative factor has been clearly identified. Nevertheless, several hypotheses have been proposed in order to explain this phenomenon. Overall, the current conception of the sundown syndrome is that of a multifactorial phenomenon, with multiple and interacting factors (resumed in Table 1) contributing to its occurrence and main phenotypic characteristics.

**Table 1:** **Factors that have been associated with the pathophysiology and clinical occurrence of sundowning among persons with dementia**.

Neurobiological factors	Degeneration of the suprachiasmatic nucleus
	Decreased melatonin production
	Disruption of circadian rhythms
	Impaired cholinergic neurotransmission
	Dysregulation of the HPA axis

Pharmacological factors	Antipsychotics
	Anticholinergics
	Antidepressants
	Hypnotics

Physiological factors	Fatigue
	Hunger
	Unmet physical or psychological needs
	Temporal changes in body temperature
	Circadian modifications of blood glucose levels
	Circadian changes in blood pressure

Medical factors	Sleep disorders
	Sensory deprivation
	Pain
	Mood disorders and fluctuations
	Cognitive deficits (e.g., agnosia)

Environmental factors	Exposure to inadequate amount of light
	Lower staff–patients ratio in residential facilities
	Lessened availability of home caregivers
	Caregiver fatigue
	Environmental overstimulation (noise and chaos)

Under a neurobiological perspective, a large body of evidence, from both animal (23) and human studies (24, 25), has been focused on the pathogenic role of primary alterations of the normal circadian rhythm (15). Circadian rhythm disorders have been linked with the involvement/alteration of the suprachiasmatic nucleus (SCN), located in the hypothalamus and considered as the major circadian pacemaker of the human body. Several studies have shown that volume, morphology, and activity of the SCN may be influenced by several factors, such as age, gender, and pathological conditions. A decrease in cell number and volume of the nucleus has been documented within the physiological aging process, especially between 80 and 100 years of age, as well as in patients with various neurodegenerative diseases (26). Neuropathological studies on AD patients have, in fact, documented relevant damages involving the SCN, mostly consisting of neuronal loss and accumulation of neurofibrillary tangles (27), while amyloid plaques are more rarely observed. The SCN of individuals with severe AD is also characterized by reactive gliosis in response to neuronal loss, with an increase in the astrocyte/neuron ratio. This damage involves both neurotensin and vasopressin neurons (26, 28). It has been thus hypothesized that sundowning (and other disruptive behaviors) may be the result of specific neuropathological abnormalities that interfere with normal circadian rhythm and behavioral regulation. An important component of circadian rhythm regulation is melatonin, a hormone secreted by the pineal gland in response to darkness, whose production and release is regulated by the SCN itself. Melatonin levels have been shown to decrease during aging and to be even more reduced in AD and other neurodegenerative diseases (29, 30). These findings have provided the rationale for the supplementation of melatonin in patients with clinical manifestations of disrupted sleep and circadian rhythm (31). The degeneration of the cholinergic system has also been indicated as a potential underlying mechanism of sundowning in AD. In fact, it has been demonstrated that the SCN receive several cholinergic projections arising from the cholinergic forebrain and brain stem nuclei. Moreover, it is sensitive to cholinergic stimulation as demonstrated by the expression of muscarinic acetylcholine receptors both in SCN neurons and astroglial cells (32). Thus, it may be hypothesized that the impaired cholinergic transmission may contribute to the disruption of circadian rhythms and the emergence of behavioral disturbances. Finally, dysregulations of the hypothalamic–pituitary–adrenal axis have been related to the pathogenesis of sundowning in AD. Specifically, patients with AD exhibiting a sundown syndrome were shown to have significantly higher cortisol levels than those without sundowning (33).

Diverse environmental determinants may contribute to the onset of sundowning. In particular, a lessened light exposure during the day, the reduced availability of caregivers or nursing staff members during late afternoon and evening, afternoon fatigue (e.g., due to intense activity during the day), the absence of a daily routine have been all associated with an overall worsening of NPS and the emergence of a sundown syndrome (8, 9, 34). In parallel, various medical conditions (e.g., pain, visual and/or hearing impairment, mood disorders) and medications (e.g., antidepressants, antipsychotics, dopaminergic therapies) may induce or exacerbate evening agitation and other behavioral disruptions (8, 15).

## Clinical Approach to Sundowning

Given the multiplicity of factors and determinants potentially involved in the emergence of sundowning, a multidimensional approach to this syndrome should be adopted. In particular, special attention should be devoted to the identification of potentially treatable/reversible underlying conditions in order to timely implement targeted interventions. As already proposed for other NPS, integrated, multistep approaches (4, 35, 36) should be adopted in screening, identifying, and managing sundowning.

In most of cases, the occurrence of sundowning is easily established through direct observation of patients (when institutionalized) or interviews to caregivers. History taking should be followed by a general physical examination, which should mostly be finalized at determining the presence of potentially contributing/precipitating somatic conditions (e.g., pain, sensory deprivation). Accordingly, a careful evaluation of potential environmental (e.g., lighting, noise levels, changes in daily routine) and iatrogenic triggers should be carried out. The diagnosis of sundowning is, thus, essentially clinical. Laboratory tests and neuroimaging studies may be performed when other causes of behavioral disruptions are suspected (e.g., delirium, cerebrovascular events). However, temporal fluctuations and patterns of symptoms, their recurrence over time, and non-acute onset should direct toward a diagnosis of a sundown syndrome (8). Moreover, the adoption of simple yet effective screening tools may support the differential diagnosis toward delirium (37). In some studies, computer devices have also been adopted to measure and quantify the diverse clinical manifestations among “sundowners” (e.g., locomotor activity, vocalizations) (38, 39). However, the use of such instruments appears difficult to be transferred in the daily clinical practice.

It is noteworthy that, to date, no dedicated tools to screen/assess sundowning has been developed and validated. Moreover, in most of clinical tools commonly adopted in the daily practice to assess the severity of NPS [e.g., the Neuropsychiatric Inventory (40)], the temporal fluctuations of disturbances are poorly considered, so that indirect information about the eventual occurrence of sundowning could not be easily deduced. The adoption of standardized instruments to evaluate NPS may also introduce some biases, being the results, and scores potentially influenced by the personal characteristics of the interviewed caregiver and poorly reliable when such tools are administered by different raters. These aspects may have probably contributed to the poor attention so far devoted to this syndrome and might have potentially produced under-recognition of the phenomenon in the clinical setting (especially among outpatients with dementia).

## Management of Sundowning

The management of sundowning represents a challenge in the clinical approach of people with dementia. First, the temporal fluctuations of symptoms as well as the heterogeneity of potential triggers/precipitants may complicate the identification and implementation of targeted and personalized interventions. Moreover, there are currently neither available guidelines nor placebo-controlled randomized trials concerning the treatment of such behavioral syndrome. Most of the available data come from case series or isolated case reports that, in most of cases, do not describe the medium- and long-term effectiveness of the adopted treatments. The treatment of sundowning may rely on the use of off-label pharmacological treatments, whose efficacy and safety profiles are still questioned and debated (4), and may result in polypharmacy and in an increased risk of psychotropic medication misuse. Based on these considerations, similarly to the other NPS, a growing consensus is being reached in considering non-pharmacological approaches as first-line treatments, limiting pharmacotherapies to non-responsive cases (35).

The studies that have so far explored the efficacy of non-pharmacological and pharmacological interventions specifically targeting sundowning in dementia are described in Table 2. Isolated case reports and researches not explicitly focusing on the sundown syndrome (i.e., more broadly referring to sleep–wake disturbances, agitation, nocturnal behavioral disruptions) were not included in the table.

**Table 2:** **Experimental studies investigating the clinical effectiveness of pharmacological and non-pharmacological therapies for the management of sundowning in dementia**.

Reference	Study design	Study sample	Intervention	Assessment	Main outcomes
**Melatonin**					
Fainstein et al. (48) Brusco et al. (49)	Open-label	41 elderly subjects (10 with Alzheimer’s disease (AD) and vascular dementia)	3 mg/day for 21 days	Daily logs of sleep and wake quality completed by caregivers	Significant decrease of sundown agitation in the 70% of demented patients. Decrease of the coefficient of variation of bed time between days 0–2 and days 19–21 of treatment (58.0 ± 24.7 vs 41.5 ± 20.9; *p* = 0.03)

Brusco et al. (49)	Retrospective	14 outpatients with AD; mean MMSE score: 14.4 ± 7.9	9 mg/day for 22–35 months	Daily logs of sleep and wake quality completed by caregivers	Remission of sundowning 12 patients; attenuation in two cases. Significant improvement of sleep quality between baseline and end of treatment (*p* < 0.01)

Cohen-Mansfield et al. (50)	Open-label	11 older nursing home residents with dementia	3 mg/day for 21 days	Daily logs of sleep and wake quality completed by nurses, Cohen-Mansfield Agitation Inventory	Significant reduction of sundown agitation between week 1 and 4 (physically non-aggressive behavior: 1.92 vs 1.46; *p* = 0.022. Verbally non-aggressive behavior: 2.30 vs 1.75; *p* = 0.028)

Cardinali et al. (31)	Open-label	45 outpatients with AD	6–9 mg/day for 4 months	Daily logs of sleep and wake quality completed by caregivers	Suppression of sundowning (regardless of the concomitant medication employed to treat cognitive or behavioral signs of AD)

Mahlberg et al. (51)	Open-label	7 AD outpatients	3 mg/day for 3 weeks	Actigraphy	Remission of sundowning in four patients; attenuation in two cases

**Light therapy**					
Satlin et al. (41)	Open-label	10 AD inpatients	2 h/day of exposure to bright light between 7:00 and 9:00 p.m. for 1 week	Clinical observation and actigraphy	Reduction of sundowning episodes

*Only studies specifically targeting sundowning were included in the table*.

### Non-Pharmacological Interventions

Individually tailored non-pharmacological approaches should be considered as the first-line therapy for sundowning. In particular, environmental modifications have been reported to be potentially beneficial to reduce sundown-related behavioral disorders. Among these, light therapy (i.e., the exposition to bright light during the afternoon/evening hours) has been observed to produce a significant reduction of sundowning episodes (41) and motor restless behaviors (42) in open-label studies conducted on patients with dementia, as well as to improve agitated behaviors in institutionalized elderly individuals (43). Nevertheless, no RCT selectively investigating the efficacy of light therapy on sundowning has been yet conducted. Accordingly, in a recent Cochrane systematic review on the topic, authors concluded that there is insufficient evidence to justify the use of bright light therapy for improving cognition, activities of daily living, sleep, challenging behaviors, and psychiatric disturbances in dementia (44). Beside the lack of robust supporting evidence, ensuring a gradual transition from daylight to artificial lighting may attenuate behavioral changes occurring in the late afternoon and is commonly suggested by health-care providers. Additional recommendations may include minimizing unnecessary noise (e.g., noise from visitors, loud speakers, banging of dishes, loud staff conversation), promoting the adherence to schedules and stable daily routines, avoiding excessive sensory stimulation during the evening (both auditory and visual), discouraging afternoon napping, and planning more challenging activities (8, 18). Finally, other non-pharmacological strategies that have been shown to produce significant benefits in the management of NPS in patients with dementia (e.g., music therapy, aromatherapy, caregiver education, multisensory stimulation) may potentially be effective also in reducing sundowning.

### Pharmacological Interventions

Most of available evidences concerning the pharmacological management of sundowning have been focused on the clinical efficacy of melatonin supplementation (theoretically supported by the documented deregulation/reduction of melatonin production in sundowners and in animal models). To date, only three RCTs have investigated the effectiveness of melatonin in reducing agitated behaviors in patients with dementia compared to placebo, also reporting inconclusive and conflicting results (45–47). Nevertheless, these studies were not specifically designed to assess sundowning, while more widely investigating changes in sleep quality, overall daytime functioning and behavior. Thus, there is no available evidence coming from *ad hoc* RCTs regarding the effects of melatonin supplementation in the treatment of sundowning in dementia. On the other hand, available open-label studies and case series (resumed in Table 2) have more consistently documented a reduction of sundowning episodes in most of patients with dementia receiving melatonin (31, 48–51). However, it should be noticed that some issues (e.g., influence of concomitant medications, severity of cognitive decline, characteristics of the living environment) were not properly addressed and may have biased the study findings and conclusions.

Cholinesterase inhibitors have repeatedly been shown to produce a meaningful decrease of behavioral disturbances in demented patients (52). Conflicting data (mainly coming from isolated case reports) exist about their potential role in positively influencing sleep disorders, circadian rhythm alterations, and sundown behavioral disruptions occurring in AD and other dementias (53, 54). Currently, there is no specific data concerning the treatment of sundowning with the N-methyl-d-aspartate receptor antagonist memantine.

Antipsychotics have been frequently indicated by physicians as the most commonly prescribed class of medications to manage sundowning (55). Nevertheless, there is limited information available in the medical literature on this particular topic, being most of RCTs focused on different NPS such as delusions, hallucinations, and agitation. Along the same lines, there is no evidence supporting the use of benzodiazepines and other hypnotics, whose use has been instead linked with a common paradoxical increase of behavioral disturbances.

## Conclusion and Future Directions

Sundowning represents a relevant and challenging manifestation of dementia, occurring in a large proportion of affected individuals and being associated with a significant social and economic burden. Increasing our knowledge on how to recognize, approach, and manage sundowning may thus consent to significantly improve the wellbeing of patients and their carers. Specifically, a greater effort is needed in order to disentangle and clarify the complex and multifaceted pathophysiological bases of this phenomenon. Moreover, dedicated screening and assessment tools should be developed and validated in order to facilitate its detection in the routine clinical practice (particularly in outpatient settings). Finally, *ad hoc* RCTs should be designed and conducted to investigate the effectiveness of non-pharmacological- and pharmacological-targeted strategies for its management.

## Author Contributions

MC and MV performed the literature search and wrote the manuscript. AT, GS, FD, and LT contributed to the conception of the article and to the review of literature. CL and GB participated to the critical appraisal of the available evidence on the topic.

## Conflict of Interest Statement

The authors declare that the research was conducted in the absence of any commercial or financial relationships that could be construed as a potential conflict of interest.
